# Association between Argyrophilic Proteins of Nucleolar Organizer Regions, Clinicomorphological Parameters, and Survival in Non-Small-Cell Lung Cancer

**DOI:** 10.1155/2014/891917

**Published:** 2014-01-02

**Authors:** Dmitriy Kobyakov, Vladimir Klimachev, Ashot Avdalyan, Igor Bobrov, Elena Bychkova, Natalia Kruglova, Aleksandr Lazarev, Elena Lushnikova, Lev Nepomnyashchikh

**Affiliations:** ^1^Molecular Diagnostic Laboratory at Altay, N.N. Blokhin ROSC (Russian Oncologic Scientific Center), RAMS (Russian Academy of Medical Sciences), Barnaul 656049, Russia; ^2^Pathologic Anatomy Chair, Altay Medical University, Barnaul 656038, Russia; ^3^General Pathologic Anatomy Laboratory, Cell Biology and Cytology Laboratory, Institute of Regional Pathology and Pathomorphology SB RAMS, Novosibirsk 630117, Russia

## Abstract

We studied argyrophilic proteins associated with nucleolar organizer regions (AgNOR) in non-small-cell cancer. We determined the area index (AI) and coefficient of variation (CV) of AgNOR. AI is associated with the key clinicomorphological parameters within the TNM system: *T* and *N* values, greatest tumor dimension up to 3 cm and more, disease stage, histogenesis, and tumor differentiation. CV is associated with *T* value, greatest tumor dimension up to 3 cm and more, histogenesis, and tumor differentiation. Survival of patients is longer in low AI or CV values versus high AI or CV values, longer in low AI and CV values (−AI/−CV type), shorter in high AI and CV values (+AI/+CV type), and intermediate in opposite AI and CV values (−AI/+CV and +AI/−CV types). Independent predictors in non-small-cell lung cancer include *N* value, greatest tumor dimension, histogenesis, and CV. Assessment of quantitative values and heterogeneity of AgNOR is important for differential diagnosis and prognosis of non-small-cell lung cancer.

## 1. Introduction

Lung cancer is a prevailing malignancy, with 80% of cases being non-small-cell cancer. Long-term treatment outcomes and survival of patients with non-small-cell cancer leave much to be desired. Therefore it is important to study morphological criteria related to the key clinicomorphological parameters of tumor and non-small-cell lung cancer patient survival and more accurate ways of predicting the course of the disease [1].

Currently evaluation of argyrophilic proteins associated with nucleolar organizer regions (AgNOR) is a generally established marker of proliferative activity and the cell cycle rate. Up to 75% of AgNOR staining is made by the two argyrophilic proteins: C23 (nucleolin) and B23 (nucleophosmin), playing a key role in ribosomal RNA synthesis. These proteins are detected in cell nuclei for the whole duration of the cell cycle, with a 1.5–3-fold increase occurring during S- and G_2_-phases [2]. Inverse relationship between quantitative amount of AgNOR and the cell cycle duration [3], doubling time of the tumor cell mass [4, 5], has been shown.

In histopathologic practice for the purpose of objectivization and standardization of AgNOR protein evaluation it was proposed to use special software for image analysis and internal staining control [6–9]. Due to heterogeneity of cell populations within one tumor it was proposed to assess variability of AgNOR in tumor cells (coefficient of variation, CV), apart from AgNOR assay [10].

Literature analysis has shown controversy of association of AgNOR with clinicomorphological parameters and survival of patients with malignant tumors [11–15]. We failed to find works specifying interdependence between the quantitative values and heterogeneity of AgNOR proteins in cell nuclei with clinicomorphological parameters and survival in non-small-cell cancer.

Considering the above, the objective of this research was to study the quantitative amount and heterogeneity of AgNOR in association with clinicomorphological parameters and survival in non-small-cell cancer.

## 2. Material and Methods

The study was performed on 243 surgical specimens of non-small-cell cancer excised in 2007–2009 at Altay Regional Oncologic Dispensary (cases of M1 and multiple tumors were excluded from the research). The mean age of patients was 59 years (35 to 75 years); the study population included 209 men and 34 women. Lobectomy was performed in 172 (70.8%) patients and pulmonectomy in 71 (29.2%) patients. Preoperative chemoradiotherapy was not used. Postoperative chemotherapy was performed in 27 of the cases (11.1%); cisplatin and etoposide were most often used. Postoperative radiation therapy was performed in 72 of the cases (29.6%), dose of 50–60 Gr most often used. Histopathological characteristics of tumors were determined according to the WHO criteria [16]. The greatest tumor dimension was measured (in cm). Fragments of pathologically intact lung tissue obtained from distant regions were used as controls in 10 cases.

Tissue fragments were fixed for 18–24 h in 10% neutral buffered formalin. After standard preparation of the surgical specimens 4 *μ*m histological slices were prepared. Specimens were stained with haematoxylin and eosin, Schiff reagent/Alcian blue, and Kreyberg stain. Cytokeratins 7 (clone SP52) and 20 (clone SP33) and high molecular weight (clone 34*β*E12) were determined immunohistochemically in an automated staining instrument Ventana XT.

In order to study AgNOR slices were stained with silver nitrate (AgNO_3_) according to a “one-step” method [17]. Before staining slices were autoclaved at 120°C for 20 minutes in a 0.01 M citrate buffer (pH = 6.0) [6, 7]. Nucleus counterstaining was not performed; slices were immersed into Canadian balm. In each case the area of AgNOR (in *μ*m^2^) was determined in nuclei of 100–120 randomly selected cells from 10–15 digital images obtained from the corresponding microscopic fields at original magnification ×1000 (lens ×100, 1.25, oil). Computer analysis of images was performed using software ImageJ 1.42. To avoid a measurement error granules of <0.1 *μ*m^2^ in size were excluded from analysis. As an internal staining standard the area of AgNOR in small lymphocyte nuclei was used [8]. AgNOR area index (AI AgNOR) was calculated as a quotient of AgNOR areas in the tumor cell and in small lymphocyte, and in each case the mean value and CV AgNOR (percent) were calculated.

Statistical analysis of data was performed using STATISTICA 6.0 software. The data obtained in the samples corresponded to the normal distribution test (Shapiro-Wilk test *W* = 0.99, *P* > 0.05); the measure of central trend in the groups was presented as the mean (M), while the measure of dispersion was presented as a standard deviation (SD). To check statistical hypotheses nonparametric methods were used, including Mann-Whitney *U* test (M-W) and Spearman's rank correlation coefficient (*r*). The total adjusted survival of patients for five years after surgery (expressed in percent) was determined using Kaplan-Meyer method, log-rank test, and Cox regression model. The reliability of the criteria obtained was assessed at *P* < 0.05.

## 3. Results and Discussion

**Figure 1:**
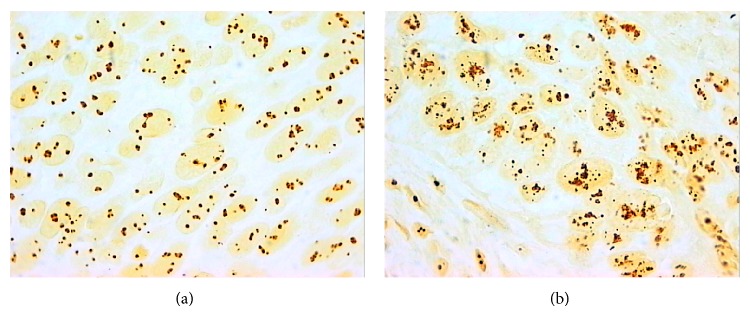
Argyrophilic proteins associated with nucleolar organizer regions (AgNOR) in nuclei of cells of moderately differentiated squamous cell lung cancer without (a) and with (b) metastases to regional lymph nodes. Silver nitrate staining, original magnification ×1000.

The result of slice staining with silver nitrate was determined as roundish black granules (AgNOR) located against the background of a brown nucleole or pale yellow nucleus (Figure 1). In nuclei of small lymphocytes one granule of silver more rarely two granules were determined. The mean area of AgNOR proteins in a small lymphocyte nucleus was equal to 1.48 (0.12) *μ*m^2^ and CV AgNOR was 19.7 (1.1)%.

Results of determination of AI AgNOR and CV AgNOR in nuclei of non-small-cell lung cancer epithelial cells in relation to the morphological parameters of tumor, as well as results of comparison with these groups, are shown in Table 1.

In non-small-cell lung cancer cells AI AgNOR was 6.52 (1.66) and CV AgNOR was 30.5 (4.6)%. In the nuclei of cells of pathologically intact epithelium of alveoli AI AgNOR was 1.31 (0.20) and CV AgNOR was 28.3 (3.1)%; in epithelium of bronchi AI AgNOR was 1.85 (0.24) and CV AgNOR was 29.3 (3.3)%. In the cells of non-small-cell lung cancer statistically significant increase of AI AgNOR and CV AgNOR was observed versus pathologically intact epithelium of alveoli and bronchi (*P* < 0.001 and *P* < 0.01). The increase of AI AgNOR and CV AgNOR in tumor cells against pathologically intact tissue supports the role of nucleolar cell mechanism in cancerogenesis.

AI AgNOR and CV AgNOR in non-small-cell lung cancer are significantly higher in group T2-3 versus T1. In primary tumors larger than 3 cm AI AgNOR and CV AgNOR were higher than in tumors smaller than 3 cm. Consequently, with the increase in greatest dimension of the tumor lesion, the increase of quantitative values of AgNOR and their heterogeneity in tumor cells were observed, testifying to the increased proliferative activity in the process of tumor growth and its heterogeneity in large-sized tumors.

Statistically significant increase of AI AgNOR in non-small-cell lung cancer with metastases to regional lymph nodes was observed versus nonmetastatic tumors (Figure 1). Consequently, the metastatic potential of non-small-cell lung cancer was associated with high quantitative values of AgNOR. However, the appearance of the metastatic potential was not accompanied by changes in intratumoral variability of AgNOR in tumor cells.

AI AgNOR in non-small-cell lung cancer was significantly higher at stages 2 and 3 versus stage 1. Therefore, at early stages of the tumor growth quantitative values of AgNOR were at the minimum versus the subsequent stages of the process. At different stages of tumor growth, intratumoral heterogeneity of AgNOR was not changed, indicating the absence of variability of AgNOR in tumor development.

AI AgNOR and CV AgNOR in non-small-cell cancer were significantly higher in squamous cell cancer versus adenocarcinoma. Therefore, there was dependence between quantitative values and heterogeneity of AgNOR and the tissue origin of the tumor.

In cells of non-small-cell lung cancer AI AgNOR and CV AgNOR were higher in moderate and low-differentiated tumors versus well-differentiated carcinoma. Qualitative values and heterogeneity of AgNOR grew depending on the degree of differentiation of non-small-cell lung cancer.

As the mean value of AI AgNOR was 6.52, the cases of AI AgNOR ≥6.52 were considered as high AI AgNOR cases (+AI), while cases of AI AgNOR <6.52 were considered low (−AI). Similarly, for CV AgNOR the mean value was 30.5%; therefore, the cases of CV AgNOR ≥30.5% were judged as high CV AgNOR (+CV), while cases of CV AgNOR <30.5 were considered low (−CV). Four types of non-small-cell lung cancer, depending on relative values of AI AgNOR and CV AgNOR were defined (Table 2).

Insignificant correlation of the key morphologic parameters of non-small-cell lung cancer was observed against the types of tumors determined: *T* value (*r* = 0.27, *P* = 0.01), greatest tumor dimension up to 3 cm and more (*r* = 0.33, *P* < 0.001), *N* value (*r* = 0.27, *P* = 0.01), stage of disease (*r* = 0.33, *P* < 0.001), histogenesis (*r* = 0.25, *P* = 0.02), and tumor differentiation degree (*r* = 0.28, *P* = 0.007).

**Figure 2:**
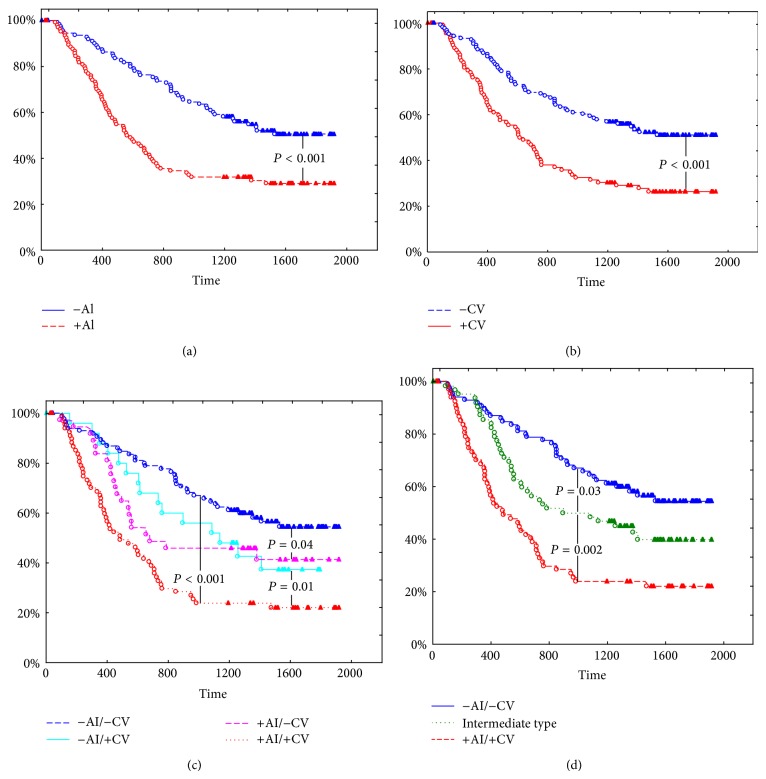
Kaplan-Meyer survival curves for patients with non-small-cell lung cancer: low and high AI AgNOR (a); low and high CV AgNOR (b); four (c) and three (d) types of tumors (according to relative values of AI AgNOR and CV AgNOR). The *X*-axis shows duration of survival (days), and the *Y*-axis shows the percentage of surviving patients.

The total adjusted survival rate of patients with non-small-cell lung cancer within five years after surgery was 40.3 ± 3.7%. Survival of non-small-cell lung cancer patients was significantly different depending on the AI AgNOR and CV AgNOR values (Table 2, Figures 2(a) and 2(b)). Consistent decrease of survival was observed in the row: Type 1, Types 2 and 3, and Type 4. Statistically significant differences in patient survival were obtained only between types 1 and 4, 1 and 3, and 4 and 2 (Table 2, Figure 2(c)). Based on the data obtained, types 2 and 3 of non-small-cell lung cancer were grouped into an “intermediate” type, where AI AgNOR and CV AgNOR in tumor cells had opposite values (−AI/+CV and +AI/−CV). Survival of patients with an “intermediate” type of tumor was significantly different from type 1 and type 4 tumors and had an intermediate value (Table 2, Figure 2(d)).

Multivariate regression analysis showed no impact of postoperative chemoradiotherapy, *T* value, degree of differentiation, AI AgNOR, and data on four or three types of tumors (according to relative values of AI AgNOR and CV AgNOR) on the non-small-cell lung cancer patient survival rates. The combination of the greatest tumor dimension and *N* value versus the stage of disease had a greater value of *χ* ^2^ (*χ* ^2^ = 103.9 and *χ* ^2^ = 91.5, resp.). Therefore, four parameters (*N* value (with or without metastases), greatest tumor dimension (up to 3 cm or more), histogenesis (adenocarcinoma or squamous cell cancer), and CV AgNOR (low or high)) were independent predictors of non-small-cell lung cancer patient survival, where the *N* value had the largest impact (Table 3). Accordingly, we studied the impact on survival of the greatest tumor dimension, histogenesis, and CV AgNOR in tumors with/without lymph nodes metastases. In non-small-cell lung cancer without metastases patient survival depended on the greatest dimension and histogenesis of tumor (*χ* ^2^ = 23.1; *P* < 0.001), and it largely depended on the CV AgNOR and the greatest tumor dimension (*χ* ^2^ = 28.3, *P* < 0.001) in patients with metastases (Table 3).

In non-small-cell lung cancer AI AgNOR and CV AgNOR had a moderate positive correlation (*r* = 0.43, *P* < 0.001), indicating the association of the changes of quantitative values of AgNOR and their heterogeneity in the tumor cells. While performing a research it is important to consider the heterogeneity of AgNOR with increased greatest tumor dimension in squamous cell cancer and in decreased differentiation, and in cases of high levels of AgNOR in tumor cells it is necessary to increase the number of the fragments of tissue being studied from different areas of the lesion.

Increased number and heterogeneity of AgNOR in non-small-cell lung cancer versus pathologically intact epithelium of bronchi and alveoli has significance for differential diagnosis when studying histopathologic lung material. Similar results were obtained in the study of epithelial neoplasms of colon, stomach, and smooth muscle uterine tumors [9, 18, 19].

AI AgNOR in non-small-cell lung cancer cells was associated with a number of clinicomorphological parameters in the TNM system: *T*, *N* values, greatest tumor dimension, stage of disease, histogenesis, and tumor differentiation. Similar conclusions were made in a number of works studying AgNOR in lung cancer [13–15]. This data may be used as additional objective criterion of differential diagnosis for differentiation of clinicomorphological parameters in the TNM system in non-small-cell lung cancer.

CV AgNOR in non-small-cell lung cancer was associated with *T* value, greatest tumor dimension, histogenesis, and tumor differentiation, reflecting the increasing heterogeneity of cell populations within tumor relative to rRNA synthesis and, probably, the rate of passing the cell cycle phases by cells. In the literature available very few studies were performed on CV AgNOR in tumor cells nuclei [20–22]. Those studies performed on specimens of breast cancer and oral squamous cell cancer also show the relation between heterogeneity (CV AgNOR) and the key clinicomorphologic parameters within the TNM system. Besides, for standardization and reproducibility of results of AgNOR research it is necessary to use the computer imaging analysis alongside the internal staining control (AI AgNOR) and heterogeneity value (CV AgNOR).

Survival of patients with non-small-cell lung cancer with −AI and −CV is significantly longer versus +AI and +CV tumors. Such interaction of the amount and heterogeneity of AgNOR with cancer patient survival was observed in other studies as well [11–15, 20–22]. According to the literature available the association between activity of AgNOR and survival of patients with carcinomas of different organs and histogenesis was significantly more frequently observed with determination of the area of AgNOR using computer imaging analysis and then in visual counting of the number of AgNOR.

Depending on the relative content of AI AgNOR and CV AgNOR four types of non-small-cell lung cancer were determined: −AI/−CV, −AI/+CV, +AI/−CV, and +AI/+CV. In our study we did not achieve a statistically significant difference between survival of patients with types −AI/+CV and +AI/−CV. That is why the two types were grouped together in a single type with a significantly different survival against −AI/−CV and +AI/+CV, showing an intermediate value. This fact shows the influence of heterogeneity of AgNOR on patient survival and specifies prognostic significance of the study.

In multivariate regression analysis *N* value, greatest tumor dimension, histogenesis, and CV AgNOR had independent influence on non-small-cell lung cancer patient survival. Numerous studies of AgNOR in malignant tumors also show that AgNOR are an independent prognostic factor [11–15, 20–22]. In non-small-cell lung cancer without metastases to lymph nodes patient survival was related to the greatest dimension and histogenesis of the tumor and in cancer with metastases to CV AgNOR and the greatest dimension of tumor. Probably, survival of patients with non-small-cell lung tumor without metastatic potential to a larger degree was dependent on with the primary tumor growth rate (local growth), while survival of patients with metastatic potential is more dependent on the heterogeneity of biosynthetic function of the tumor cell population.

In conclusion, the study of the number and heterogeneity of AgNOR has differential-diagnostic and prognostic significance in non-small-cell lung cancer.

## 4. Conclusions

1. AI AgNOR in non-small-cell lung cancer is associated with the key clinicomorphological parameters within the TNM system: *T*, *N* values, greatest tumor dimension up to 3 cm and more, disease stage, histogenesis, and tumor differentiation. CV AgNOR is associated with *T* value, greatest tumor dimension up to 3 cm and more, histogenesis, and tumor differentiation. This data may be used as additional objective criterion of differential diagnosis for differentiation of clinicomorphological parameters in the TNM system in non-small-cell lung cancer.
2. Non-small-cell lung cancer patient survival is longer in low AI AgNOR or CV AgNOR tumors versus high AI AgNOR or CV AgNOR tumors.
3. Non-small-cell lung cancer patient survival is longer in low AI and CV AgNOR (−AI/−CV type), shorter in high AI and CV AgNOR (+AI/+CV type), and intermediate in opposite AI and CV AgNOR values (−AI/+CV and +AI/−CV types).
4. Independent predictors in non-small-cell cancer include *N* value, greatest tumor dimension, histogenesis, and CV AgNOR. In non-small-cell lung cancer without metastases to lymph nodes patient survival is determined by the greatest tumor dimension and histogenesis, but it is determined by the CV AgNOR and greatest tumor dimension when metastases are present.

## Figures and Tables

**Table 1:** AI AgNOR and CV AgNOR in non-small-cell lung cancer.

Parameter	Number of cases (abs. (%))	AgNOR			
		AI	M-W	CV	M-W
Primary tumor					
T 1	70 (29)	5.89 ± 1.49	<0.001	29.5 ± 4.5	0.006
T 2-3	173 (71)	6.81 ± 1.68		31.1 ± 4.6	
Greatest dimension					
<3 cm	104 (43)	5.84 ± 1.54	<0.001	29.3 ± 4.0	<0.001
≥3 cm	139 (57)	7.05 ± 1.58		31.5 ± 4.8	
Lymph nodes					
N 0	157 (65)	6.27 ± 1.59	<0.001	30.3 ± 4.5	0.1
N 1–3	86 (35)	7.06 ± 1.70		31.2 ± 4.8	
Stage					
I	139 (57)	6.23 ± 1.62	<0.001	30.4 ± 4.5	0.5
II-III	104 (43)	6.99 ± 1.64		30.9 ± 4.7	
Histogenesis					
Adenocarcinoma	111 (46)	6.05 ± 1.78	<0.001	29.8 ± 4.7	0.02
Squamous cell	132 (54)	6.96 ± 1.46		31.2 ± 4.4	
Differentiation					
High	63 (26)	5.90 ± 1.54	<0.001	29.4 ± 3.7	0.02
Moderate-low	180 (74)	6.76 ± 1.66		31.0 ± 4.8	

**Table 2:** AI AgNOR and CV AgNOR and five-year total adjusted survival in non-small-cell lung cancer.

Parameter	Number of cases (abs. (%))	Five-year total adjusted survival
AI AgNOR		
Low	125 (51)	49.8 ± 5.2
High	118 (49)	28.6 ± 4.7
CV AgNOR		
Low	135 (56)	50.2 ± 4.9
High	108 (44)	25.8 ± 4.9
Type of tumor according to the AI AgNOR and CV AgNOR		
Type 1: −AI/−CV	94 (39)	53.2 ± 6.0
Type 2: −AI/+CV	30 (12)	38.5 ± 10.0
Type 3: +AI/−CV	42 (17)	38.7 ± 9.1
Type 4: +AI/+CV	77 (32)	20.7 ± 5.2
“Intermediate” type	72 (29)	38.4 ± 6.8

**Table 3:** Cox regression analysis and prognostic factors in non-small-cell lung cancer.

Prognostic factor	*β*	Standard error	*P*
*N* value	1.63	0.20	<0.001
Greatest tumor dimension	0.96	0.21	<0.001
Histogenesis	0.29	0.09	0.002
CV AgNOR	0.66	0.19	<0.001
Without metastases			
Greatest tumor dimension	1.13	0.32	<0.001
Histogenesis	0.43	0.14	0.002
CV AgNOR	0.22	0.28	0.4
With metastases			
Greatest tumor dimension	0.92	0.29	0.001
Histogenesis	0.23	0.13	0.08
CV AgNOR	1.02	0.29	<0.001
